# Azithromycin and Ceftriaxone Differentially Activate NLRP3 in LPS Primed Cancer Cells

**DOI:** 10.3390/ijms23169484

**Published:** 2022-08-22

**Authors:** Gulcin Tezcan, Mohammad Alsaadi, Shaimaa Hamza, Ekaterina E. Garanina, Ekaterina V. Martynova, Gulshat R. Ziganshina, Elina R. Farukshina, Albert A. Rizvanov, Svetlana F. Khaiboullina

**Affiliations:** 1 Institute of Fundamental Medicine and Biology, Kazan Federal University, 420008 Kazan, Russia; 2 Department of Fundamental Sciences, Faculty of Dentistry, Bursa Uludag University, Bursa 16059, Turkey

**Keywords:** azithromycin, ceftriaxone, Nod-like receptor protein 3 (NLRP3), inflammasome, cancer

## Abstract

Background: Cancer patients are prescribed antibiotics, such as macrolides and lactamides, for infection treatment. However, the effect of these antibiotics on NLRP3 activation remains largely unknown. Method: Lung cancer (A549) and prostate cancer (PC3) cell lines were primed with lipopolysaccharide (LPS) to activate NLRP3 transcription. Cells were then treated with azithromycin (Az) or ceftriaxone (Cf). NLRP3 activation was analyzed by qPCR, Western blot, and ELISA. Cell growth and viability were assessed by real-time cell analysis and Annexin V expression. Levels of 41 cytokines were also analyzed using a multiplex assay. Results: LPS-Az activated transcription of *NLRP3*, *Pro-CASP-1*, and *Pro-IL-1β* in A549 cells, while failing to upregulate *NLRP3* and *Pro-IL-1β* in PC3 cells. LPS-Az decreased the secretion of pro-inflammatory cytokines while it induced the pro-angiogenic factors in A549 and PC3 cells. In contrast, LPS-Cf suppressed the expression of NLRP3-associated genes, NLRP3 protein expression, the inflammatory cytokine secretion in A549 and PC3 cells. LPS-Az and LPS-Cf had a limited effect on cell growth and viability. Discussion: Our data suggest that Cf could suppress LPS induced NLRP3, which should be considered when selecting antibiotics for cancer treatment. In contrast, the effect of Az on LPS primed NLRP3 and the inflammatory cytokines production appears to depend on the cancer cell origin. Therefore, these data indicate that considerations are required when selecting Az for the treatment of cancer patients.

## 1. Introduction

Infection-induced inflammation could facilitate tumor development [1]. Emerging evidence indicates that inflammation is one of the most crucial risk factors that contributes to the failure of cancer treatment [2,3]. One of several mechanisms identified as regulating inflammation is the Nod-like receptor protein 3 (NLRP3) inflammasome [4]. This inflammasome releases an active form of Caspase-1 (CASP1), which, subsequently, cleaves interleukin 1β (IL-1β), a potent pro-inflammatory cytokine [4]. Within the family of inflammasome proteins, NLRP3 is primarily shown to be involved in cancer development and progression [5,6]. It was suggested that, by supporting long-term inflammation, this inflammasome could contribute to cancer progression [7]. This is illustrated by the overexpression of NLRP3 inflammasome, which promotes aggressive tumor features, such as enhanced proliferation, survival, and metastasis [8,9,10,11,12,13,14,15,16,17]. Our previous study also provided evidence of NLRP3 activation being associated with cancer cell growth [18]. We have also demonstrated that the effect of NLRP3 activation on cancer cell survival depends upon inflammasome mediated cytokine release [18]. Our previous data indicate that some antibiotics, such as nigericin, activate the inflammasome, support inflammation, and have a pro-tumorigenic effect [18]. In contrast, other antibiotics, such as doxycycline, could oppose inflammasome activation and have an inhibitory effect on cancer cell growth [19]. Therefore, our data emphasize the importance of testing the effect of antibiotics on inflammasome activation to avoid selection of antimicrobial agent supporting inflammation and tumor progression when used to treat cancer patients.

Patients with stomach, lung, and prostate malignancies where bacterial infection is one of the risk factors often have long-term antibiotic treatment as part of their therapy protocol [20,21,22]. Many antibiotics are used for treatment of cancers because of their anti-proliferative, pro-apoptotic, and anti-epithelial-mesenchymal transition effects [23,24,25,26]. However, long-term administration of antibiotics in cancer patients could have undesirable consequences, such as promoting chronic inflammation [27], impairing the metabolism of healthy tissue [28], reducing activity of the cell division checkpoint inhibitors [29], and weakening immune defense [20,27].

Some antibiotics have the potential to affect NLRP3 activation by targeting host cell mitochondria [19,30]. Mitochondria are the primary source of cellular reactive oxygen species (ROS) [31] which promote NLRP3 activation [32]. Antibiotics, including lactamides and macrolides, are capable of increasing ROS production [33,34,35]. Recent studies have identified that azithromycin (Az), a macrolide, can also cause mitochondrial toxicity and overproduction of ROS [36]. However, the effect of azithromycin on NLRP3 in cancer cells remain unknown. It should be noted that resistance to cefoxitin, a lactamide antibiotic, is linked to high IL-1β serum levels in methicillin-resistant Staphylococcus aureus (MRSA) infected patients [37]. Targeting NLRP3 was shown to avert the progression of infections with Gram-positive and Gram-negative bacteria [38]. These findings indicate that the NLRP3 induction could be targeted by antibiotics to treat infectious diseases [38]. However, the indiscriminate use of antibiotics such as lactamides could lead to antibiotic resistance [37]. It appears that the pathogenesis of this resistance could involve inflammasome mechanisms, as a study by Müller et al. reported that exacerbation of MRSA immunopathology by lactamides is IL-1β-dependent [37]. It should be noted that these antibiotics are commonly used for the treatment of bacterial infection in cancer patients [20,39,40]. As NLRP3 activation and IL-1β secretion could promote tumor growth and contribute to chemotherapy resistance [41], considerations should be made before using lactamides. Although lactamides and macrolides are frequently used for treating a bacterial infection in cancer patients [20,39,40], our understanding of these antibiotics’ effects on NLRP3 activation remains limited.

In this study, we aimed to investigate the impact of Az, a macrolide [42], and ceftriaxone (Cf), a lactamide [43], antibiotics on NLRP3 expression in LPS-primed cancer cells. Previously, we identified that NLRP3 activation was low in the lung cancer cell line, A549, whilst it was high in the prostate cancer cell line, PC3 [18]. Therefore, in this study, we selected these cell lines to investigate the effect of Az and Cf on NLRP3. We demonstrated the effect of LPS-Az on transcription of NLRP3 pathway genes, such as NLRP3, Pro-CASP-1, and Pro-IL-1β, as well as on the expression of NLRP3 protein. Additionally, the effect of LPS-Az on cancer cell viability and pro-inflammatory cytokine secretion was investigated. We have found that although Az and Cf suppress NLRP3 protein in PC3 cells, Az did not affect NLRP3 expression in A549 cells. Additionally, Az and Cf failed to inhibit the proliferation and vitality of LPS primed PC3 and A549 cells.

## 2. Results

**Figure 1:**
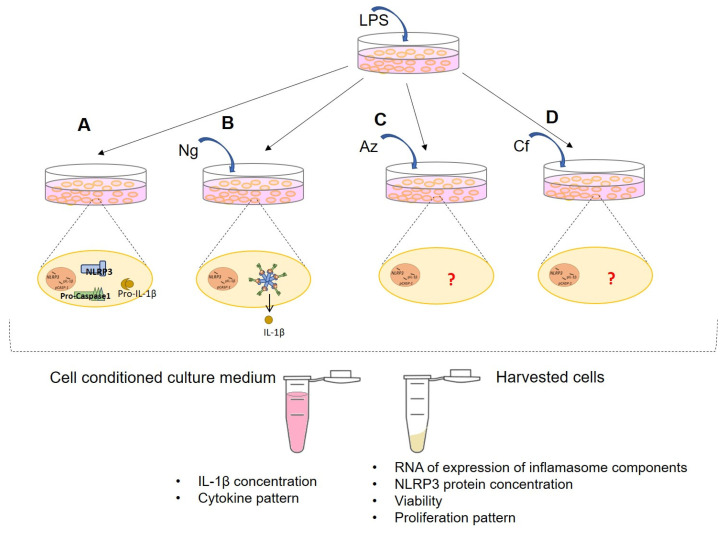
Experimental design of treatment schedules for A549 and PC3 cells. (**A**). LPS-only treatment for 3 h to prime NLRP3. (**B**). LPS-Ng treatment for 24 h are used as a positive control to activate the NLRP3 inflammasome complex. (**C**,**D**). The experimental test groups are treated with (**C**) Az or (**D**) Cf for 24 h after pretreatment with LPS for 3 h.

A priming signal by a pathogen-associated molecular pattern (PAMP) such as LPS is required for the transcriptional activation of *NLRP3* and *pro–IL-1β* genes [44,45,46]. A pore-forming bacterial toxin, Nigericin, serves as a second stimulus, inducing potassium efflux, which triggers NLRP3 activation and the formation of an active multiprotein inflammasome complex [47]. Therefore, in this study, A549 and PC3 cells were treated with LPS (1 µg/mL; 3 h) to induce *NLRP3* and *pro-IL-1β* RNA synthesis. Ng (20 µm) was added to initiate inflammasome activation and assembly [18,19]. Antibiotics Az (150 µm) and Cf (100 µg/mL) were added 3 h post LPS treatment. A schematic of the experimental design is shown in Figure 1.

### 2.1. Cf and Az Suppress NLRP3 Inflammasome

**Figure 2:**
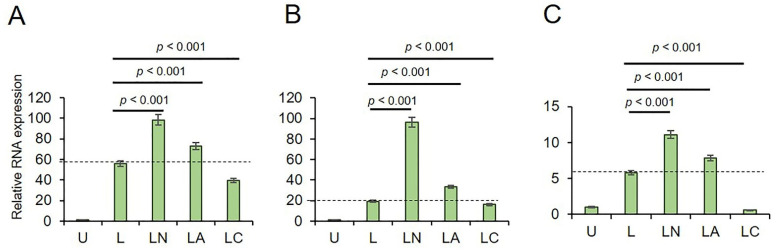
The effect of LPS-Az and LPS-Cf on RNA levels of NLRP3 associated genes in A549 cells. (**A**) NLRP3, (**B**) Pro-CASP1, (**C**) Pro-IL-1β. U: Untreated, L: LPS, LN: LPS-Ng, LA: LPS-Az, LC: LPS-Cf. *p* values were calculated using one-way ANOVA and Tukey’s post-hoc tests (n = 3). Dotted line indicates RNA expression in LPS primed A549 cells. Data is presented as Mean ± SD.

In A549 cells, LPS only increased the transcription of *NLRP3* (*p* < 0.001), *pro-CAPS1* (*p* < 0.001), and *Pro-IL-1β* (*p* < 0.001) genes (Figure 2A–C, and Table S1) as compared to untreated controls. As expected, LPS-Ng further increased the expression of these genes (*p* < 0.001). When cells were treated with LPS-Az, the expression of *NLRP3*, *pro-CAPS1*, and *Pro-IL-1β* genes remained upregulated compared to untreated controls and LPS only (*p* < 0.001). However, this upregulation had lesser efficacy than LPS-Ng (*p* < 0.001). In contrast to LPS-Az, LPS-Cf upregulated *NLRP3* and *Pro-CASP1* transcription compared to the untreated control (*p* < 0.001). Expression of the *Pro-IL-1β* gene remained unaffected in A549 cells treated with LPS-Cf compared to the untreated control (*p* < 0.001). It should be noted that LPS-Cf activation of *NLRP3* and *pro-CAPS1* transcription was less effective as compared to that in LPS only (*p* < 0.001) and in the LPS-Ng groups (*p* < 0.001). These data indicate that genes associated with the NLRP3 pathway are activated in A549 cells treated with LPS and LPS-Ng, commonly used for inflammasome induction and complex formation. Our data demonstrate that macrolide Az activated, while lactamide Cf had a limited effect on the activation of NLRP3 pathway genes.

**Figure 3:**
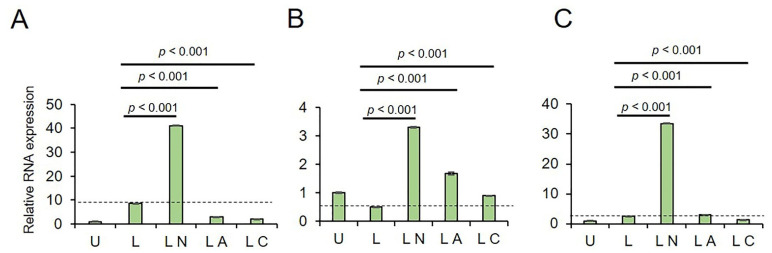
The effect of LPS-Az and LPS-Cf on RNA levels of NLRP3 associated genes in PC3 cells. (**A**) NLRP3, (**B**) Pro-CASP1, (**C**) Pro-IL-1β. U: Untreated, L: LPS, LN: LPS-Ng, LA: LPS-Az, LC: LPS-Cf. *p* values were calculated using one-way ANOVA and Tukey’s post-hoc tests (n = 3). Dotted line indicates RNA expression in LPS primed PC3 cells. Data is presented as Mean ± SD.

In PC3 cells, LPS-only upregulated the expression of only *NLRP3* and *Pro-IL-1β* genes, while it had a limited effect on pro-CAPS1 gene transcription compared to untreated cells (Figure 3A–C, and Table S1). LPS-Ng substantially activated the transcription of *NLRP3*, *pro-CAPS1*, and *Pro-IL-1β* genes compared to untreated controls (*p* < 0.001; Table S1) and LPS only. In contrast, LPS-Az failed to upregulate *NLRP3* and *Pro-IL-1β*, while only *pro-CAPS1* transcription was increased compared to untreated controls and LPS-only. Lactamide in combination with LPS (LPS-Cf) had a limited effect on the expression of *NLRP3*, *pro-CAPS1*, and *Pro-IL-1β*. These data suggest that lactamide Cf has a limited effect on the transcription of NLRP3 pathway genes. In contrast, macrolide Az has a cell-type specific effect on these genes’ transcription and NLRP3 activation.

**Figure 4:**
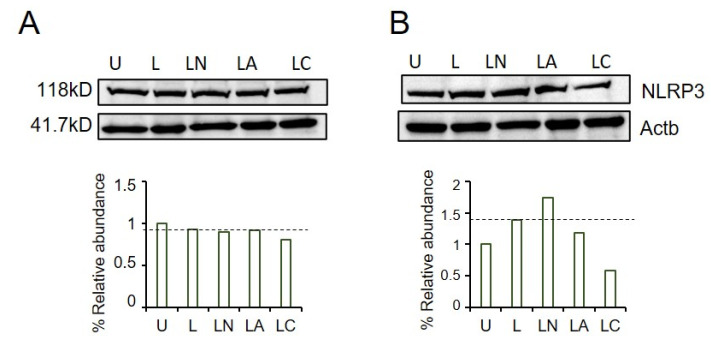
The effect of LPS-Az and LPS-Cf on NLRP3 protein in (**A**) A549 and (**B**) PC3 cells. U: Untreated, L: LPS, LN: LPS-ng, LA: LPS-Az, LC: LPS-Cf. Dotted line indicates the relative NLRP3 protein expression in LPS primed cells. Western blots were visualized using ChemiDoc XRS Plus (Bio-Rad, Hercules, CA, USA). Protein levels were quantified using ImageJ v1.53s software (National Institutes of Health, Bethesda, MD, USA).

Although the transcription of NLRP3 was upregulated in LPS-only, LPS-Ng and LPS-Az treated A549 cells, the protein expression remained unaffected (Figure 4A). In contrast, LPS-only and LPS-Ng increased NLRP3 levels in PC3 cells compared to untreated controls (Figure 4B). The expression of inflammasome protein in these cells treated with a macrolide antibiotic, LPS-Az, demonstrated the upregulation of NLRP3 compared to untreated controls. However, this upregulation had lesser efficacy as compared to LPS-only. Expression of inflammasome protein was lower in lactamide, LPS-Cf, compared to untreated controls. These data demonstrate that the effect of macrolide Az on NLRP3 protein expression could vary depending on cancer cell type. In contrast, lactamide Cf attenuates NLRP3 protein expression in both cell lines.

**Figure 5:**
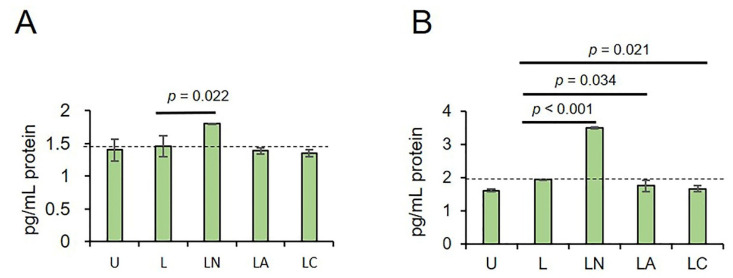
The effect of LPS-Az and LPS-Cf on IL-1β secretion in A549 (**A**) and PC3 cells (**B**). U: Untreated, L: LPS, LN: LPS-ng, LA: LPS-Az, LC: LPS-Cf. *p* values were calculated using one-way ANOVA and Tukey’s post-hoc tests. Dotted line indicates the secreted IL-1β in LPS primed cells. IL-1β levels were analyzed using Interleukin-1 beta-EIA-BEST kit (VECTOR-BEST, Novosibirsk, Russia). Results were captured using a TECAN Infinite 200 Pro fluorimeter (Grödig, Austria) at a wavelength of 450 nm and quantified using a standard curve. Each ELISA was completed in technical duplicates.

To demonstrate the formation of an active inflammasome and caspase 1, the levels of NLRP3 product, IL-1β, were analyzed in cells supernatants. In A549 cells, an increased IL-1β secretion was detected only in cells treated with LPS-Ng compared to LPS only (*p* = 0.022, Figure 5A) and untreated controls (*p* = 0.008, Table S2). In PC3 cells, IL-1β secretion was higher in LPS-Ng compared to untreated controls (*p* < 0.001, Table S2). Macrolide and lactamide, LPS-Az (*p* = 0.034), and LPS-Cf (*p* = 0.021) suppressed the release of IL-1β as compared to LPS-only (Figure 5B), indicating that macrolide Az and lactamide Cf reduce the production of an active form of IL-1β.

### 2.2. Az and Cf Did Not Affect Cell Viability

**Figure 6:**
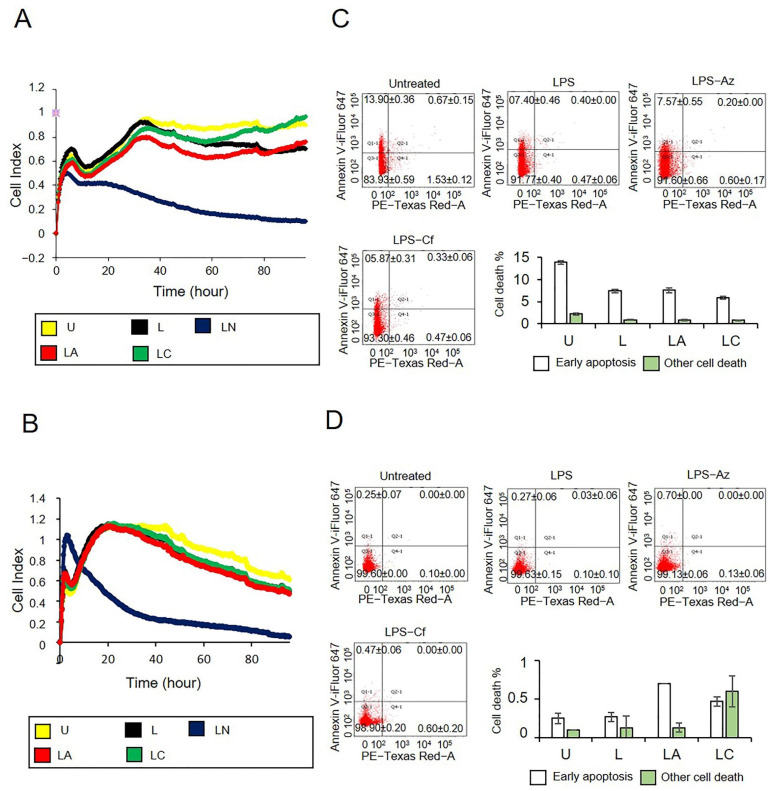
The effect of azithromycin and ceftriaxone induced activation of NLRP3 on proliferation and cell viability of A549 and PC3 cells. (**A**) The effect of LPS-azithromycin and LPS-ceftriaxone treatments on A549 cell proliferation; (**B**) PC3 cell proliferation; (**C**) A549 cell viability; and (**D**) PC3 cell viability. Adjusted *p*-values were calculated using Independent Samples Kruskal–Wallis Test. U: Untreated, L: LPS, LA: LPS-Az, LC: LPS-Cf.

Next, the effect of LPS-only, LPS-Ng, LPS-Az and LPS-Cf treatment on A549 and PC3 cell growth kinetics was analyzed in real time for 96 h. Cell proliferation kinetics indicated that LPS-Az and LPS-Cf did not affect cell proliferation, while LPS-Ng substantially reduced the cell growth in both A549 and PC3 cells (Figure 6A,B). Additionally, LPS-Az and LPS-Cf had a limited effect on A549 or PC3 cell viability (Figure 6C,D, Table S3). These data demonstrate that NLRP3 activation reduces cell viability and proliferation. In contrast, macrolide Az and lactamide Cf do not cause NLRP3-dependent cell death.

### 2.3. Az and Cf Differently Affect Pro-Inflammatory Cytokine Secretion

**Figure 7:**
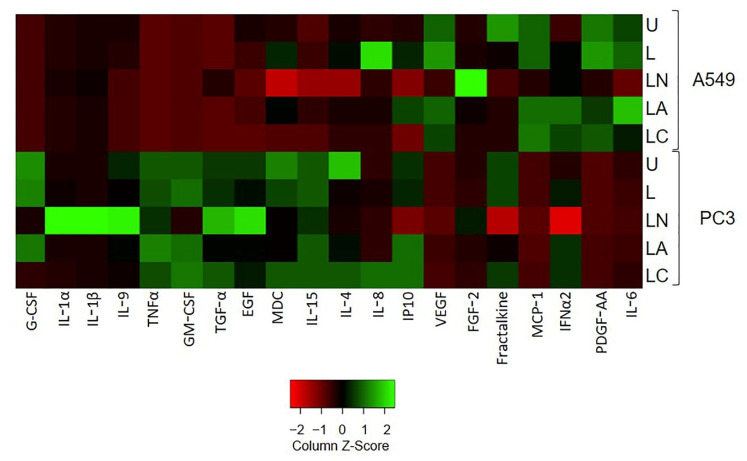
The effect of LPS-Az and LPS-Cf on cytokine secretion in A549 and PC3 cells. The heat map describes changes in the levels of cytokine secretion patterns in A549 and PC3 cells after LPS, LPS-Ng, LPS-Az and LPS-Cf treatments. The cytokines concentrations that were not detected were excluded from the heatmap graphs. U: Untreated, L: LPS, LN: LPS-Ng, LA: LPS-Az, LC: LPS-Cf.

The level of 41 secreted cytokines was measured 24 h after treatment with LPS-only, LPS-Ng, LPS-Az, and LPS-Cf. The secretion levels of IL-1α, IL-1β, IL-4, IL-8, IL-9, TGF-α, EGF, G-CSF, IP10, TNFα, and GM-CSF were lower in untreated A549 cells compared to untreated PC3 cells. Among these cytokines, the secretion levels of pro-inflammatory IL-4, IL-8, G-CSF, and IP10/CXCL10 were induced upon LPS-only treatment while they were reduced by LPS-Nig in A549 cells, indicating the higher pro-inflammatory capacity of PC3 cells as compared to A546 cells. In contrast, after LPS-Az treatment, higher IL-8 but lower IL-4, G-CSF, and IP10 secretions were detected compared to LPS-only-treated A549 cells. In addition, the secretion of IL-1α, TGF-α, EGF, TNFα, and GM-CSF was not affected by LPS-only treatment in A549 cells (Figure 7). However, among these cytokines, pro-inflammatory IL-1α and TGF-α were induced by LPS-Ng compared to untreated and LPS-only-treated A549 cells, while they were not affected by LPS-Az. (Figure 7). In contrast, pro-inflammatory EGF, TNFα, and GM-CSF were decreased in both LPS-Ng-, and LPS-Az-treated A549 cells (Figure 7, Table S4).

In PC3 cells, LPS-only treatment did not affect any of these cytokines, except that it caused elevation of IL-1α. However, LPS-Ng induced the secretion of IL-1α, IL-8, IL-9, TGF-α, EGF, and GM-CSF. In contrast, while LPS-Az treatment reduced IL-1α, TGF-α, and EGF, it did not affect the secretion of IL-8, and GM-CSF compared to untreated and LPS-only-treated PC3 cells.

The secretion levels of Fractalkine, FGF-2, IFNa2, PDGF-AA, VEGF, MCP-1, and IL-6 were higher in untreated A549 cells than in untreated PC3 cells. Among these cytokines, the secretion levels of FGF2, PDGF-AA, VEGF, and IL-6 were induced upon LPS-only treatment in A549 cells. In addition, LPS-Ng induced FGF2 while it reduced PDGF-AA, VEGF, and IL-6 compared to untreated and LPS-only-treated A549 cells. In contrast, LPS-Az induced FGF2 and VEGF while it reduced PDGF-AA and IL-6 in A549 cells (Figure 7, Table S4)

In PC3 cells, while Fractalkine, FGF-2, IFNa2, PDGF-AA, VEGF, MCP-1, and IL-6 was not affected by LPS-only treatment, LPS-Ng reduced their secretion, except that it caused elevation of FGF2. However, LPS-Az treatment reduced the secretion of Fractalkine and MCP-1 while it induced the secretion of FGF-2, IFNa2, PDGF-AA, and VEGF (Figure 7, Table S4).

Among the cytokines, which were detected in lower levels in untreated A549 cells compared to untreated PC3 cells (IL-1α, IL-1β, IL-4, IL-8, IL-9, TGF-α, EGF, G-CSF, IP10, TNFα, GM-CSF), LPS-Cf decreased the secretion of IL-4, IL-8, G-CSF and IP10, which were induced upon LPS-only treatment and IL-1α and TGF-α, which were not affected by LPS-only treatment compared to untreated A549 cells. In PC3 cells, where LPS-only treatment did not affect these cytokines, LPS-Ng increased IL-8, IL-9, TGF-α, and EGF, while LPS-Cf did not affect their secretion compared to untreated and LPS-only-treated cells (Figure 7, Table S4).

Among the cytokines, which were detected in higher levels in untreated A549 cells compared to untreated PC3 cells (Fractalkine, FGF-2, IFNa2, PDGF-AA, VEGF, MCP-1, and IL-6), LPS-Cf decreased the secretion of FGF2, PDGF-AA, VEGF and IL-6 compared to untreated and LPS-only-treated A549 cells. In PC3 cells, while LPS-Cf reduced the secretion of FGF2 and VEGF, it induced the secretion of IFNa2, MCP-1, and IL-6.

These data indicate that macrolide Az and lactamide Cf attenuate the inflammatory milieu in cells when NLRP3 is activated. However, macrolide Az also induces cytokines such as IL-18, VEGF and FGF2, which have the potential to promote angiogenesis and metastasis [48,49,50].

## 3. Discussion

Our data demonstrate that macrolide, Az, and lactamide Cf antibiotics could regulate inflammasome activation after priming of cancer cells with LPS, a microbial endotoxin. When A549 cells, previously shown to have limited NLRP3 activation capacity, were treated with LPS-Az or LPS-Cf, only Az synergized with endotoxin stimulating *NLRP3*, *pro-CAPS1* and *Pro-IL-1β* genes expression. It appears that, despite the activation of transcription, inflammasome proteins and functional complexes were not formed, as IL-1β release remained unchanged. In PC3 cells, characterized by high inflammasome activation, macrolide Az only increased the transcription of *pro-CAPS1*. Our data demonstrate that the expression of NLRP3 was not affected in A549 cells treated with LPS-Az as compared to LPS-only. In contrast, LPS-Az inhibited inflammasome protein expression in PC3 cells. Still, it should be noted that the NLRP3 level in LPS-only- and LPS-Az-treated A549 cells was slightly lower than in untreated cells. This could be explained by the previously published observation that LPS priming produces less mitochondrial damage in A549 as compared to that in PC3 cells [18]. This reduced damage to mitochondria was suggested to explain a lower level of activating NLRP3 in A549 as compared to in PC3 cells. The role of mitochondrial damage in inflammasome activation is supported by the Az mechanism of action which specifically binds to the 50S subunit of the bacterial ribosome, homologous to the 39S eukaryotic mitochondrial ribosome [51]. Therefore, the Az’s effect on NLRP3 expression in cancer cells could vary depending on the degree of mitochondrial damage inflicted by this antibiotic [52]. On the other hand, lactamides, including Cf, target the cell wall structural integrity by disrupting the synthesis of the peptidoglycan [53]. Additionally, some studies have suggested that lactamides block eukaryotic DNA synthesis [54,55,56]. This could explain the limited effect of lactamide, Cf, on the transcription and protein secretion of inflammasome-related genes in both cell lines. Collectively, these data indicate that macrolide and lactamide antibiotics could differ in their capacity to support the inflammasome activation and formation of a functional complex in A549 and PC3 cancer cells.

Caspase dependent enhancement of TRAIL-induced apoptosis in cancer cells was suggested as the mechanism of Az anti-tumor efficacy [57]. Our data corroborate Az-mediated activation of caspase in cancer cells as we showed that LPS-Az induced the expression of *CASP1* RNA as compared to LPS-only. It should be noted that, in contrast to the previous study when only Az was used [57], we did not find cancer cell death when Az was combined with LPS. This could be explained by LPS-induced inflammation which was shown to be capable of supporting cancer cell survival [58]. Therefore, we suggest that LPS-priming of NLRP3 could activate pro-inflammatory cytokines production which reduces the apoptotic effect of Az.

Macrolide Az was shown to reduce LPS-induced IL-1β expression, which is controlled by NLRP3 [59,60,61]. Similar to these studies, we showed that LPS-Az reduces IL-1β RNA expression and the secretion of A549 and PC3 cells. LPS-Az reduced NLRP3 RNA expression and protein synthesis of PC3 cells, which were shown to have high NLRP3 activation capacity [18]. Interestingly, in A549 cells, characterized by a lower ability to activate NLRP3 [18], LPS-Az had a limited effect on NLRP3. Therefore, our data suggest that LPS-Az has an inhibitory effect on NLRP3 in cancer cells with a high capacity for inflammasome activation.

We have also found that LPS-Az decreased IL-4, G-CSF, and IP10/CXCL10 secretion in A549 cells while it induced IFNα2 and reduced IL-1α, TGF-α, and EGF secretions in PC3 cells. In contrast, an opposite secretion pattern of these cytokines was demonstrated in cells treated with LPS-Ng, which form a functional NLRP3 complex. These cytokines could contribute to cancer immune surveillance. The elevated tumor secretion of IL-4 was found to play a role in the modification of cancer cell differentiation and growth and resistance to apoptosis [62]. Tumor cells producing high levels of G-CSF demonstrate an aggressive phenotype and they were reported as more difficult to treat [63,64,65,66]. The IP10/CXCL10 was shown to mediate interactions between tumor and stromal cells and to organize leukocyte trafficking and angiogenesis which results in the induction of metastasis [67]. IL-1α could activate NFκB–regulated genes coding for proteins contributing to the tumor inflammatory microenvironment [68,69]. TGF-α is known as a marker for malignancy due to its tumor transformation ability of normal cells using the EGF pathway [70,71]. In contrast, IFNα2 could increase the expression of major histocompatibility complex (MHC) molecules, essential for immune recognition of tumors [72,73]. These data suggest that macrolide Az-mediated NLRP3 suppression could interfere with the maintenance of the inflammatory milieu required for the survival of cancer cells. However, it should be noted that LPS-Az induced the secretion of a pro-inflammatory cytokine IL-8 in A549 and pro-angiogenic factors, VEGF and FGF2 in A549 and PC3 cells. IL-8 was shown to induce cancer growth, enhance angiogenesis and promote metastasis [48]. An elevated production of FGF-2 was shown to support a malignant cancer phenotype and chemotherapy resistance by activation Ras-MAPK and PI3K pathways [49]. VEGF has been recognized as the key mediator of cancer angiogenesis which is essential for cancer development and growth [50]. In addition, based on the results of a clinical trial [74], The US Food and Drug Administration (FDA) warned that there is an increased risk of tumor relapse in cases with long-term use of azithromycin [75]. Therefore, these data suggest that, although macrolide Az mediated NLRP3 suppression could interfere with maintenance of the inflammatory milieu required for the survival of cancer cells, its potential to increase angiogenesis and metastasis should be considered with long-term use in cancer patients.

Lactam antibiotics could also target inflammasomes. It was reported that Cf, a third-generation cephalosporin lactam, used after priming cells with high concentration of calcium, could activate NLRP3 [76]. However, it remained unclear whether the inflammasome activation was Cf associated, or if it was the result of calcium-sensing receptors stimulation which, by reducing intracellular cAMP levels, could also activate NLRP3 [77]. Our data showed that LPS-Cf decreased *NLRP3* and *pro-IL-1β* RNA expression and reduced these proteins production as compared to LPS-only. Therefore, we suggest that Cf could suppress NLRP3 in cancer cells when LPS is used as a priming agent. It appears that NLRP3 activation by Cf could require priming factors other than LPS. The high levels of calcium found in the tumor microenvironment [76,77] could be an alternative priming factor.

Similar to macrolide LPS-Az, lactamide LPS-Cf decreased the secretion of pro-inflammatory cytokines, such as IL-4, G-CSF, IP10/CXCL10 and TGFα in A549 cells which has a low NLRP3 activation capacity. LPS-Az failed to reduce the secretion of these cytokines in PC3 cells, which has a high NLRP3 activation capacity. However, considering the NLRP3 activation-dependent induction of these cytokines in PC3 cells treated with LPS-Ng, our findings showed that the levels of these cytokines were not elevated as a result of LPS-Az treatment. In contrast to LPS-Az, LPS-Cf decreased the secretion of FGF2 and VEGF as compared to LPS-only treated A549 and PC3 cells. Therefore, these data indicate that Cf mediated NLRP3 suppression could also be combined with the reduced inflammation, which is essential to maintain the pro-tumorigenic microenvironment.

In conclusion, for the first time, we analyzed the effect of the macrolide Az and lactamide Cf antibiotics on NLRP3 activation in A549 and PC3 cancer cells. We have found that, upon LPS priming, macrolide Az activated the transcription of inflammasome genes in A549 cells, while it was not effective in PC3 cells. In contrast, lactamide Cf had a limited effect on these genes’ transcription in A549 cells. In LPS primed PC3 cells, only Az could activate Pro-CASP1 gene transcription, while no effect of Cf was demonstrated on all of the inflammasome genes studied. While assessing protein expression and IL-1β synthesis, we found that LPS priming failed to produce inflammasome proteins and IL-1β secretion in A549 cells. LPS priming of PC3 cells increased NLRP3 protein production, while IL-1β secretion was reduced. We have also shown that Az and Cf had a limited effect on LPS-primed A549 and PC3 cells’ vitality. We found that cytokine production differed in Az- and Cf-treated A549 and PC3 primed cells. Our data suggest that, while Cf reduces NLRP3 activation in both cell lines, the effect of Az could vary depending on cell line. These data provide evidence suggesting that considerations are required when selecting antibiotics for the treatment of bacterial infection in cancer patients. Although Az and Cf antibiotics did not affect LPS primed cancer cell viability in our study in vitro, an in vivo analysis of the long-term effects of these antibiotics on tumor growth is warranted.

## 4. Materials and Methods

### 4.1. Cell Lines and Reagents

A549, a lung cancer cell line, and PC3, a prostate cancer cell line, were obtained from the American Type Culture Collection (ATCC; Rockville, MD, USA). Cells were maintained (5% CO_2_, 37 °C) using Dulbecco’s Modified Eagle’s Medium-F12 (PanEco, Moscow, Russia), supplemented with 10% fetal bovine serum (HYCLONE, Logan, UT, USA), 2 mm L-glutamine and 1 mm sodium pyruvate (PanEco, Moscow, Russia).

Lipopolysaccharides (LPS) from Escherichia coli O111:B4 (L4391), nigericin (Ng) (N7143), Az (PHR1088) and Cf (C5793) were purchased from Sigma (St. Louis, MO, USA).

### 4.2. Real Time-qPCR

Total RNA was extracted using Trizol (Sigma, St. Louis, MO, USA) [78]. RNA concentration and integrity was determined by the 260:280 and 260:230 ratios measured by NanoDrop 2000 spectrophotometer (Thermo Scientific, Wilmington, DE, USA). Total RNA (500 nm) was used for cDNA synthesis using RevertAid First Strand cDNA Synthesis Kit (Thermo Fisher Scientific, Inc., Waltham, MA, USA). The expression of genes, *NLRP3* (*NLRP3*), *pro-caspase-1* (*pro-CASP1*) and *pro-IL-1β*, was analyzed using qPCR. Primers are summarized in Table 1 [21,79]. The RNA input was normalized using a housekeeping gene, *Actin β* (*ACTB*). The threshold cycle (Ct) for RNA expression was determined using the CFX384 Touch™ Real-Time PCR Detection System (Biorad, CA, USA). Fold changes of Ct values were calculated using the 2^−ΔCt^ method. 

Primer sequences were designed using the GenScript DNA Sequencing Primers Design Tool (Piscataway, NJ, USA; https://www.genscript.com/tools/dna-sequencing-primer-design (accessed on 25 July 2019)) [79].

### 4.3. Immunoblotting

Total proteins were extracted using the radioimmunoprecipitation assay (RIPA) buffer and were quantified using the Pierce BCA Protein Assay Kit (Thermo Fischer Scientific, USA). Proteins were separated in 8–12% gradient polyacrylamide gels and transferred onto Immuno-Blot PVDF membranes (Biorad, Hercules, CA, USA). Membranes were blocked (5% non-fat milk, 1 h) and incubated (overnight, 4 °C) with primary antibody: human anti-NLRP3 (1:500, Invitrogen, IL, USA). Membranes were washed (3×, 10 min with PBS containing 0.1% Tween 20) and incubated (2 h, room temperature (RT)) with the secondary antibody, anti-rabbit IgG (1:1000, Santa Cruz Biotechnology, Heidelberg, Germany). A mouse anti-human Actin β -HRP conjugated antibody (1:1000, Sigma, St. Louis, MO, USA) was used as an internal control for protein load. Blots were developed (Clarity Western ECL Substrate; Bio-Rad, Hercules, CA, USA) and visualized using ChemiDoc XRS Plus (Bio-Rad, Hercules, CA, USA). Protein levels were quantified using ImageJ v1.53s software (National Institutes of Health, Bethesda, MD, USA).

### 4.4. Enzyme-Linked Immunosorbent Assay (ELISA)

IL-1β levels were assessed using Interleukin-1 beta-EIA-BEST kit (VECTOR-BEST, Novosibirsk, Russia). Briefly, 100 µL of the cell-free medium was added to anti-human IL-1β antigen pre-coated wells for 2 h at RT. Blocked (5% bovine serum albumin (BSA)), wells were incubated with anti-human-HRP conjugated antibodies for 1 h at RT. Washed (3×; 0.5% Tween20 in PBS), wells were incubated with 100 µL of substrate solution in the dark for 30 min at RT. The reaction was ended by adding a stop solution. Results were collected using an Infinite 200 Pro fluorometer (Life Sciences-Tecan, Grödig, Austria) at a wavelength of 450 nm and quantified using a standard curve. Each ELISA was completed in technical duplicates.

### 4.5. Real-Time Cell Proliferation Analysis

A549 and PC3 cells (5 × 10^3^) were plated onto an E-Plate 16 (ACEA Biosciences, San Diego, CA, USA) in the standard medium. The electrical impedance was measured every 15 min over 96 h to monitor the cell proliferation using the xCELLigence biosensor cell analysis system (ACEA Biosciences, San Diego, CA, USA).

### 4.6. Cell Viability Assay

The viability of cells was assessed using APC Annexin V Apoptosis Detection Kit, following the manufacturer’s instructions (Sony Biotechnology, San Jose, CA, USA). Briefly, cells were harvested in 100 µL of the annexin labeling solution (2% annexin-V–APC, 0.1 µg/mL propidium iodide (PI)) in Annexin V binding buffer and incubated in the dark for 15 min, PT. The flow cytometry analysis was performed using BD FACSAria III Flow Cytometer (BD Biosciences, San Diego, CA, USA). Data were processed with the FlowJo software package (FlowJo LLC, Ashland, OR, USA). Experiments were conducted in three technical repeats. Cells positive for annexin-V-APC-only were identified as early-apoptotic, whereas cells positive for both, Annexin V and PI or PI-only, were counted as late-apoptotic or non-apoptotic, respectively [80].

### 4.7. Multiplex Cytokine Analysis

Cytokine levels were determined by using magnetic bead suspension array MILLIPLEX^®^ Human Cytokine/Chemokine Magnetic Bead Panel–Premixed 41 Plex, following the manufacturer’s directions (EMD Millipore, Billerica, MA, USA). Cell-conditioned medium (50 µL) was analyzed by collecting a minimum of 50 beads per analyte. Median fluorescence intensities were collected by MAGPIX analyzer (Luminex, Austin, TX, USA and data were analyzed using MilliPlex Analyst v.5.1analysis software (MiraiBio, San Bruno, CA, USA). The standard curves for each cytokine were plotted using the standards provided with the kit. Measured cytokine levels were presented as a heatmap using a web-based tool (http://www.heatmapper.ca/ (accessed on 26 December 2021)) [81].

### 4.8. Statistical Analysis

Statistical analysis was performed using the one-way ANOVA model with Tukey’s post hoc tests and linear regression analysis using IBM SPSS Statistics for Windows, Version 20.0 (IBM Corp., Armonk, NY, USA). Data are presented as mean  ±  SE. Statistical significance was set at a *p*-value < 0.05.

## Figures and Tables

**Table 1:** Primer set sequences used for the RT-qPCR analyze.

Primers		
*NLRP3*	F:	5′-ATGAGTGCTGCTTCGACATC-3′
	R:	5′-TTGTCACTCAGGTCCAGCTC-3′
*Pro-CASP1*	F:	5′-TGCCTTTCTTCTGGTCAGTG-3′
	R:	5′-TGCTGAGGTGAAGGAGAGAA-3′
*Pro-IL-1β*	F:	5′-TCAGCACCTCTCAAGCAGAA-3′
	R:	5′-GGACTCTCTGGGTACAGCTC-3′
*ACTB*	F:	5′-GACAGGATGCAGAAGGAGATTACT-3′
	R:	5′-TGATCCACATCTGCTGGAAGGT-3′

## Data Availability

All data generated or analyzed during this study are included in this published article. The data that support the findings of this study are available from the corresponding author upon request.

## Supplementary Materials

The following supporting information can be downloaded at: https://www.mdpi.com/article/10.3390/ijms23169484/s1.
