# Supplementary material for: Azithromycin and Ceftriaxone Differentially Activate NLRP3 in LPS Primed Cancer Cells
Source: Int J Mol Sci. 2022 Aug 22;23(16):9484. doi: 10.3390/ijms23169484 (PMC9409354; doi:10.3390/ijms23169484)
Supplement: Supplementary file 1 [file ijms-23-09484-s001.zip › ijms-1865135-supplementary.pdf]

Azithromycin and Ceftriaxone differentially activate NLRP3 in LPS Primed Cancer Cells

Supplementary Tables:

Table S1. The effect of azithromycin and ceftriaxone on mRNA expression of *NLRP3* and downstream genes.

Cell type	Gene	Comparison between	Mean Difference	Std. Err.	p	95% Confidence Interval Lower Bound	Upper Bound
A549	NLRP3	U-L	-54.94	0.01	<0.001	-54.98	-54.90
		U-LN	-97.45	0.01	<0.001	-97.49	-97.41
		U-LA	-71.66	0.01	<0.001	-71.70	-71.62
		U-LC	-38.44	0.01	<0.001	-38.48	-38.40
		L-LN	-42.50	0.01	<0.001	-42.54	-42.46
		L-LA	-16.71	0.01	<0.001	-16.75	-16.67
		L-LC	16.51	0.01	<0.001	16.47	16.55
		LN-LA	25.79	0.01	<0.001	25.75	25.83
		LN-LC	59.01	0.01	<0.001	58.97	59.05
		LA-LC	33.22	0.01	<0.001	33.18	33.26
A549	pro-IL-1β	U-L	-4.80	0.01	<0.001	-4.84	-4.76
		U-LN	-10.07	0.01	<0.001	-10.11	-10.04
		U-LA	-6.80	0.01	<0.001	-6.83	-6.76
		U-LC	0.43	0.01	0.001	0.39	0.46
		L-LN	-5.27	0.01	<0.001	-5.31	-5.24
		L-LA	-1.99	0.01	<0.001	-2.03	-1.96
		L-LC	5.23	0.01	<0.001	5.19	5.27
		LN-LA	3.28	0.01	<0.001	3.24	3.32
		LN-LC	10.50	0.01	<0.001	10.46	10.54
		LA-LC	7.22	0.01	<0.001	7.19	7.26
A549	Pro-CASP1	U-L	-18.31	0.01	<0.001	-18.33	-18.28
		U-LN	-95.23	0.01	<0.001	-95.25	-95.20
		U-LA	-32.38	0.01	<0.001	-32.41	-32.35
		U-LC	-14.95	0.01	<0.001	-14.97	-14.92
		L-LN	-76.92	0.01	<0.001	-76.95	-76.89
		L-LA	-14.07	0.01	<0.001	-14.10	-14.05
		L-LC	3.36	0.01	<0.001	3.33	3.39
		LN-LA	62.85	0.01	<0.001	62.82	62.87
		LN-LC	80.28	0.01	<0.001	80.25	80.31
		LA-LC	17.43	0.01	<0.001	17.40	17.46
PC3	NLRP3	U-L	-7.66	0.06	<0.001	-7.86	-7.46
		U-LN	-40.14	0.06	<0.001	-40.34	-39.94
		U-LA	-1.98	0.06	<0.001	-2.18	-1.78
		U-LC	-1.14	0.06	<0.001	-1.34	-0.94
		L-LN	-32.48	0.06	<0.001	-32.68	-32.28
		L-LA	5.68	0.06	<0.001	5.48	5.88
		L-LC	6.52	0.06	<0.001	6.32	6.72
		LN-LA	38.16	0.06	<0.001	37.96	38.36
		LN-LC	39.00	0.06	<0.001	38.80	39.20
		LA-LC	0.84	0.06	<0.001	0.64	1.04
PC3	pro-IL-1β	U-L	-1.57	0.02	<0.001	-1.64	-1.51
		U-LN	-32.55	0.02	<0.001	-32.61	-32.49
		U-LA	-1.92	0.02	<0.001	-1.98	-1.86
		U-LC	-0.41	0.02	<0.001	-0.47	-0.35
		L-LN	-30.98	0.02	<0.001	-31.04	-30.92
		L-LA	-0.35	0.02	<0.001	-0.41	-0.29
		L-LC	1.16	0.02	<0.001	1.10	1.22
		LN-LA	30.63	0.02	<0.001	30.57	30.69
		LN-LC	32.14	0.02	<0.001	32.08	32.20
		LA-LC	1.51	0.02	<0.001	1.45	1.57
PC3	Pro-CASP1	U-L	0.50	0.02	<0.001	0.43	0.58
		U-LN	-2.30	0.02	<0.001	-2.37	-2.22
		U-LA	-0.68	0.02	<0.001	-0.75	-0.60
		U-LC	0.11	0.02	0.006	0.03	0.18
		L-LN	-2.80	0.02	<0.001	-2.87	-2.72
		L-LA	-1.18	0.02	<0.001	-1.25	-1.10
		L-LC	-0.40	0.02	<0.001	-0.47	-0.32
		LN-LA	1.62	0.02	<0.001	1.55	1.70
		LN-LC	2.40	0.02	<0.001	2.33	2.48
		LA-LC	0.78	0.02	<0.001	0.71	0.86

*P-value calculated using One Way Anova and Tukey Test. P values less than 0.05 were considered significant. U: Untreated, , L: LPS, LN: LPS-Ng, LA: LPS-Az, LC: LPS-Cf, n=3.

Table S2. The effect of azithromycin and ceftriaxone on IL-1β secretion

Cell line	Comparison between	Mean Difference	Std. Err.	p	95% CI	
					Lower Bound	Upper Bound
A549	U-L	-0.06	0.09	0.961	-0.36	0.24
	U-LN	-0.41	0.09	0.008	-0.70	-0.11
	U-LA	0.01	0.09	1.000	-0.29	0.31
	U-LC	0.05	0.09	0.984	-0.25	0.34
	L-LN	-0.35	0.09	0.022	-0.64	-0.05
	L-LA	0.07	0.09	0.926	-0.23	0.37
	L-LC	0.11	0.09	0.766	-0.19	0.40
	LN-LA	0.42	0.09	0.007	0.12	0.72
	LN-LC	0.45	0.09	0.004	0.15	0.75
	LA-LC	0.03	0.09	0.995	-0.26	0.33
PC3	U-L	-0.33	0.07	0.007	-0.58	-0.09
	U-LN	-1.89	0.07	<0.001	-2.13	-1.64
	U-LA	-0.26	0.07	0.032	-0.50	-0.02
	U-LC	-0.05	0.07	0.954	-0.29	0.19
	L-LN	-1.55	0.07	<0.001	-1.79	-1.31
	L-LA	0.19	0.05	0.034	0.01	0.36
	L-LC	0.28	0.07	0.021	0.04	0.52
	LN-LA	1.62	0.07	<0.001	1.38	1.86
	LN-LC	1.84	0.07	<0.001	1.59	2.08
	LA-LC	0.21	0.07	0.093	-0.03	0.45

*P-value calculated using One Way Anova and Tukey Test. P values less than 0.05 were considered significant and showed in bold. U: Untreated, L: LPS, LN: LPS-Ng, LA: LPS-Az, LC: LPS-Cf, n=2.

Table S3. The effect of azithromycin and ceftriaxone on cell death.

Cell	Cell death	df	Test statistics	p (within group)	Comparison between	p (pair-wase)
A549	Early apoptosis (Q1)	3.00	9.46	0.024	U-L	0.537
					U-LA	1.000
					U-LC	0.130
					L-LA	1.000
					L-LC	1.000
					LA-LC	0.537
A549	Other cell death (Q2 + Q4)	3	6.92	0.074		
PC3	Early apoptosis (Q1)	3	9.16	0.027	U-L	1.000
					U-LA	0.085
					U-LC	0.905
					L-LA	0.059
					L-LC	0.884
					LA-LC	1.000
PC3	Other cell death (Q2 + Q4)	3	6.73	0.081		

*P-value calculated using Independent Samples Kruskal Wallis Test. P values less than 0.05 were considered significant. U: Untreated, L: LPS, LN: LPS-Ng, LA: LPS-Az, LC: LPS-Cf

Table S4: The effect of azithromycin and ceftriaxone on cytokine releasing pattern of A549 and PC3 cells.

	A549					PC3				
	U	L	LN	LA	LC	U	L	LN	LA	LC
EGF	4.78	3.6	<2.22↓	2.95	<2.22↓	8.92	6.82	16.11	6.34	7.06
FGF-2	144.76	221.28	3527	348.03	76.29	68.97	49.43	788.49	131.89	31.82
Eotaxin	<2.27↓	<2.27↓	<2.27↓	<2.27↓	<2.27↓	<2.27↓	<2.27↓	<2.27↓	<2.27↓	<2.27↓
TGF-a	<1.63↓	<1.63↓	3.49	<1.63↓	<1.63↓	6.72	6.33	11.6	4.9	7.91
G-CSF	143.8	173.47	49.3	131.6	135.69	16534	15394	3220	15207	>18601↑
Flt-3L	<2.49↓	<2.49↓	<2.49↓	<2.49↓	<2.49↓	<2.49↓	<2.49↓	<2.49↓	<2.49↓	<2.49↓
GM-CSF	42.82	41.5	15.49	39.75	41.72	3370	3597	880.79	3621	3801
Fractalkine	97.37	50.83	36.41	43.85	45.63	75.66	75.66	3.09	50.83	72.77
IFNa2	14.6	18.71	18.71	26.1	23.64	15.42	20.36	2.58	22	22
IFN-y	<1.51↓	<1.51↓	<1.51↓	<1.51↓	<1.51↓	<1.51↓	<1.51↓	<1.51↓	<1.51↓	<1.51↓
GRO	>11613↑	>11613↑	1161	>11613↑	>11613↑	>11613↑	>11613↑	>11613↑	>11613↑	>11613↑
IL-10	NAN	NAN	NAN	NAN	NAN	NAN	NAN	NAN	NAN	NAN
MCP-3	<1.82↓	<1.82↓	<1.82↓	<1.82↓	<1.82↓	<1.82↓	<1.82↓	<1.82↓	<1.82↓	<1.82↓
IL12p40	<1.77↓	<1.77↓	<1.77↓	<1.77↓	<1.77↓	<1.77↓	<1.77↓	<1.77↓	<1.77↓	<1.77↓
MDC	<0.82↓	3.87	<0.82↓	1.08	<0.82↓	<0.82↓	1.08	<0.82↓	<0.82↓	2.53
IL12p70	NAN	NAN	NAN	NAN	NAN	NAN	NAN	NAN	NAN	NAN
PDGF-AA	591.57	684.61	115.97	425.81	509.15	16.57	16.09	<1.71↓	28.24	21.84
IL-13	<1.56↓	<1.56↓	<1.56↓	<1.56↓	<1.56↓	<1.56↓	<1.56↓	<1.56↓	<1.56↓	<1.56↓
PDGF-AB/BB	<16.00↓	<16.00↓	<16.00↓	<16.00↓	<16.00↓	<16.00↓	<16.00↓	<16.00↓	<16.00↓	<16.00↓
IL-15	2.06	2.73	<1.30↓	3.06	1.42	2.84	2.73	<1.30↓	3.4	3.4
sCD40L	<1.92↓	<1.92↓	<1.92↓	<1.92↓	<1.92↓	<1.92↓	<1.92↓	<1.92↓	<1.92↓	<1.92↓
IL-17A	<1.70↓	<1.70↓	<1.70↓	<1.70↓	<1.70↓	<1.70↓	<1.70↓	<1.70↓	<1.70↓	<1.70↓
IL1Ra	<3.98↓	<3.98↓	<3.98↓	<3.98↓	<3.98↓	<3.98↓	<3.98↓	<3.98↓	<3.98↓	<3.98↓
IL1a	<1.55↓	<1.55↓	8.59	<1.55↓	<1.55↓	14.25	23.05	372.39	12.8	1.93
IL-9	7.17	5.84	<1.67↓	<1.67↓	2.12	15.34	9.8	42.33	11.3	8.74
IL-1b	<1.40↓	<1.40↓	1.8	<1.40↓	<1.40↓	<1.40↓	<1.40↓	9.28	<1.40↓	<1.40↓
IL-2	<1.57↓	<1.57↓	<1.57↓	<1.57↓	<1.57↓	<1.57↓	<1.57↓	<1.57↓	<1.57↓	<1.57↓
IL-3	<1.53↓	<1.53↓	<1.53↓	<1.53↓	<1.53↓	<1.53↓	<1.53↓	<1.53↓	<1.53↓	<1.53↓
IL-4	10.71	15.05	<2.03↓	13.36	8.75	20.21	17.1	12.69	12.69	18.13
IL-5	<2.00↓	<2.00↓	<2.00↓	<2.00↓	<2.00↓	<2.00↓	<2.00↓	<2.00↓	<2.00↓	<2.00↓
IL-6	27.41	32.09	<1.82↓	46.73	21.3	10.07	8.72	6.01	8.23	10.52
IL-7	<1.84↓	<1.84↓	<1.84↓	<1.84↓	<1.84↓	<1.84↓	<1.84↓	<1.84↓	<1.84↓	<1.84↓
IL-8	2437	3135	261.27	3374	2615	>7775↑	>7775↑	6395	>7775↑	>7775↑
IP10	21.41	26.46	4.43	20.88	17.91	49.87	22.19	20.88	25.97	36.16
MCP-1	5687	5682	1629	6142	6219	801.21	736.66	120.1	617.88	769.41
MIP-1a	<2.12↓	<2.12↓	<2.12↓	<2.12↓	<2.12↓	<2.12↓	<2.12↓	<2.12↓	<2.12↓	<2.12↓
MIP-1b	<1.79↓	<1.79↓	<1.79↓	<1.79↓	<1.79↓	<1.79↓	<1.79↓	4.17	<1.79↓	<1.79↓
Rantes	<6.13↓	<6.13↓	<6.13↓	<6.13↓	<6.13↓	<6.13↓	<6.13↓	<6.13↓	<6.13↓	<6.13↓
TNFa	<1.65↓	1.78	<1.65↓	<1.65↓	<1.65↓	41.43	40.38	32.74	52.47	39.33
TNF-b	<1.93↓	<1.93↓	<1.93↓	<1.93↓	<1.93↓	<1.93↓	<1.93↓	<1.93↓	<1.93↓	<1.93↓
VEGF	430.16	554.29	94.61	437.24	360.59	63.34	60.19	23.72	82.83	59.22

Cytokine secretion is presented as pg/ml. **U:** Untreated, **L:** LPS, **LN:** LPS-Ng, **LA:** LPS-Az, **LC:** LPS-Cf

↓: Lower than minimum detectable concentration, ↑: Higher than maximum detectable concentration
